# Effects of corn particle size on nutrient utilization in pigs evaluated under optimal and heat stress conditions

**DOI:** 10.1007/s11250-018-1711-7

**Published:** 2018-09-20

**Authors:** Won Yun, Min Ho Song, Ji Hwan Lee, Chang Hee Lee, Seo Young Oh, Woo Gi Kwak, Doo Wan Kim, Hyeun Bum Kim, Jin Ho Cho

**Affiliations:** 1 Department of Animal Science, Chungbuk National University, Cheongju, Chungbuk 28644 Republic of Korea; 2 Department of Animal Science and Biotechnology, Chungnam National University, Daejeon, 34134 Republic of Korea; 3 Swine Division, National Institute of Animal Science, Rural Development Administration, Cheonan, 31000 Republic of Korea; 4 Department of Animal Resource and Science, Dankook University, Cheonan, 31116 Republic of Korea

**Keywords:** High temperature, Thermal response, Nutrient digestibility, Growing pig

## Abstract

The effects of corn particle size on nutrient digestibility and energy utilization in pigs were determined under optimal (experiment 1, 25 ± 1 °C) or heat stress (experiment 2, 37 ± 1 °C) conditions. In Exp. 1 and 2, five experimental diets were tested using a 5 × 5 Latin square design involving five barrows (Landrace × Yorkshire × Duroc, average initial body weight of 30 ± 1 kg and 45.0 ± 1.8 kg, respectively, in individual metabolic cages). Dietary treatments were as follows: 200-, 300-, 400-, 600-, 800-μm corn particle sizes obtained by mesh screens. Under optimal thermal conditions, digestibility of dry matter (DM) and crude fiber (CF) from 200-μm diet was higher (*P* < 0.05) compared to that from the 300-μm and 400-μm diets. The digestibility of crude protein (CP) and ether extract (EE) was the highest (*P* < 0.05) at the 200-μm particle size. The apparent total tract digestibility of energy was significantly higher (*P* < 0.05) on the 200-μm diet. Under heat stress, digestibility of CF when corn was ground to 600 μm was higher (*P* < 0.05) compared to 300 and 400 μm. Digestibility of NDF and ADF was the highest (*P* < 0.05) at 600-μm corn particle size. In conclusion, grinding corn to 200-μm corn particles had a positive effect on DM, CP, EE, and CF under optimal thermal condition, while the 600-μm corn particle size had positive effects on digestibility of CF, NDF, and ADF than 200-μm corn particle size under heat stress.

## Introduction

In modern pig farming, feed accounts for a major part of production costs. Accordingly, it is necessary to improve the processing of feeds in order to maximize nutrient availability. In this regard, reducing the particle size of cereal grains by cracking, crimping, rolling, or grinding via mechanical force can increase the surface area and improve digestibility (Ohh et al. 1983). This improvement in nutrient digestibility and gain/feed ratio is attributed to an increase in the area of contact with digestive enzymes (Kim et al. 2000; Hancock et al. 2000). However, grains that are too finely ground can cause stomach ulcers in growing-finishing pigs (Nielsen and Ingvartsen 2000). Weaning pigs fed with larger particle size, which might allow more time for mastication and digestion in the mouth, show slower growth than pigs fed a finer particle-sized diet (Kim et al. 2005). Similarly, previous research has shown that a reduction in the particle size of grain from coarse to fine can increase gain/feed ratio (Lawrence 1983). Further, reducing the particle size of cereal grains to 600 μm has been shown to result in greater nutrient digestibility, rate of growth, and lactation performance, and decreased fecal excretion of nutrients, when compared with the effects of diets of coarser particle sizes between 900 and 1000 μm (Wondra et al. 1995b). The growth performance of pigs can also be influenced by temperature. In this regard, Quiniou et al. (2000) reported that the average daily feed intake (ADFI) of pigs was reduced by heat stress at high ambient temperature. Consistent with these findings, the growth performance of growing finishing pigs in an optimal environment was shown to be superior to that in a high-temperature environment (Le Bellego et al. 2002). Thus, the objective of the present study was to investigate the effects of reducing the particle size of corn feed on the growth performance and nutrient digestibility of pigs maintained under heat stress and optimal environmental conditions.

## Materials and methods

### Experiment design and housing

The protocol for the two experiments was approved by the Institutional Animal Care and Use Committee of Chungbuk National University, Cheongju, Republic of Korea.

**Fig. 1:**
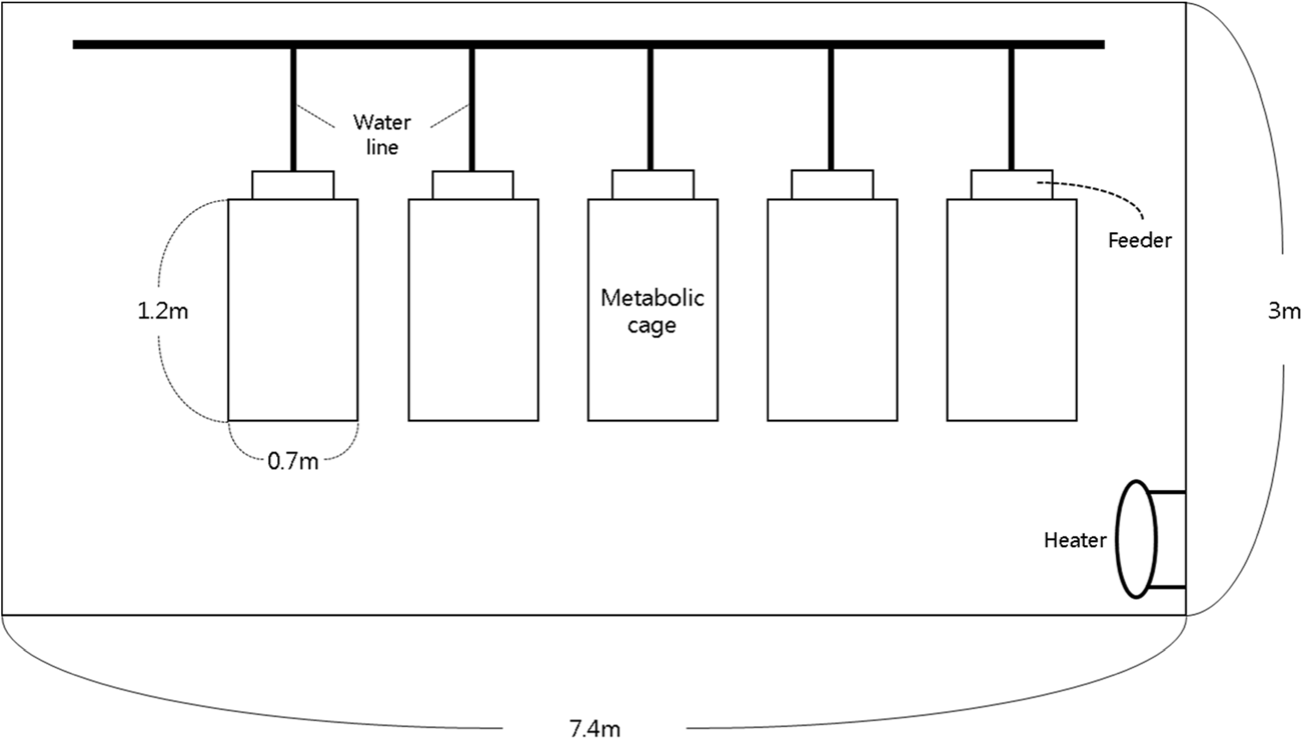
Schematic drawing of experimental room

In experiment 1, a total of five crossbred (Duroc × Landrace × Yorkshire) barrows were randomly allotted five diets over five periods in a 5 × 5 Latin square design. The pigs (average initial body weight of 30.0 ± 1.1 kg) were individually housed in 1.2 m × 0.7 m × 0.96 m stainless steel metabolism cages in an environmentally controlled room (Fig. 1). The experimental condition was conducted at 24 ± 2 °C temperature with 85 ± 1.3% relative humidity and air speed was 0.25 ± 0.02 m/s.

In experiment 2, a total of five crossbred (Duroc × Landrace × Yorkshire) barrows were allotted five diets over five periods in a 5 × 5 Latin square design. The pigs (average initial body weight of 45.0 ± 1.8 kg) were individually housed in 1.2 m × 0.7 m × 0.96 m stainless steel metabolism cages in an environmentally controlled room (Fig. 1). The experiment was conducted at 37 ± 1 °C temperature with 85 ± 1.3% relative humidity and air speed was 0.25 ± 0.02 m/s. The temperature and relative humidity were based on average temperatures found in piggeries in summer seasons of South Korea.

### Diets and feeding

Table 1 shows the nutrient contents of the main ingredients used in this experiment. Five diets were formulated using various sizes of corn particles (Table 1). The other ingredients except for corn had mean particle size of 1000 μm. These other ingredients except for corn were the same for all treatments. The daily feed allowance was adjusted to 2.7 times the maintenance requirement for DE (2.7 × 110 kcal of DE/kg BW^0.75^; NRC 1998). The allowance was divided into two equal parts and fed at 08:00 and 17:00 h. The diets were mixed with water in a ratio of 1:1 (wt/wt) before feeding. Pigs had free access to water during the experiment.

**Table 1:** Chemical composition of the basal diets (as-fed basis)

Ingredients	Percent
Corn	54.13
Soybean meal	30.45
Wheat	3.00
Canola meal	2.00
Soybean oil	4.29
Molasses	3.00
Limestone	0.63
Calcium phosphate	1.47
Lysine	0.34
Methionine	0.08
Threonine	0.03
Choline Cl	0.03
Mineral premix	0.10
Vitamin premix	0.20
Salt	0.25
Total	100
Analyzed chemical composition	
Dry matter (%)	98.1
Crude protein (%)	18.5
Ether extract (%)	6.7
Crude ash (%)	5.0
NDF (%)	8.5
ADF (%)	3.5
Gross energy (MJ/kg)	20.4

### Sampling and analysis

The pigs were weighed individually at the beginning of each period and the amount of feed supplied for each period was recorded, as well as any residual feed quantity. Each experimental period consisted of a 4-day adaptation period followed by a 3-day collection period to collect feces and urine. The feces and urine were collected by total collection method. Feces were collected immediately when the feces appeared in the metabolism cages, kept in plastic bags, and stored at − 20 °C. Urine was collected once a day into buckets containing 50 mL of 6 mol/L HCl that were placed under the metabolism cages. The collected urine was weighed and stored at − 20 °C. The collection of feces and urine was conducted according to the methods described by Song et al. (2003). Fecal samples were dried in a forced air oven and ground through a 1-mm screen, and thoroughly mixed before a subsample was collected for chemical analysis. Diets and feces were analyzed for dry matter (AOAC, 1990), crude protein (AOAC, 1990), and crude fiber (AOAC, 1990). The gross energy of diets, feces, and urine was analyzed using an adiabatic oxygen bomb calorimeter (Parr Instruments, Moline, IL). The content of nitrogen in the urine was also analyzed (AOAC, 1990).

### Statistical analysis

The data for effects of various particle sizes of corn on the apparent total tract digestibility (ATTD) of fiber, dry matter, protein, energy, and available energy of the test diets were subjected to an analysis of variance using PROC GLM of SAS (Statistical Analysis System 9.1, SAS Institute, Cary, NC, USA).

## Results

### Exp. 1. Under optimal thermal conditions

Table 2 presents effects of various corn particle sizes on the apparent total tract digestibility of nutrients. Intakes of DM, CP, EE, CF, CA, NDF, and ADF were different among treatments (*P* < 0.05). Pigs fed the 300- and 400-μm diets excreted higher concentrations of DM and CP than pigs fed the 200-μm corn diet (*P* < 0.05). The EE excreted from the 200-μm treatment was significantly lower than that from other treatments (*P* < 0.05). Content of CA in excretion from 200-μm treatment was significantly higher than that from the 800-μm treatment. Apparent total tract digestibility of DM and CF from 200-μm diet was higher than that from 300- and 400-μm diets, and apparent total tract digestibility of CP and EE from 200-μm diet was higher than that of other treatments. Apparent total tract digestibility of CA for 400-, 600-, and 800-μm treatments was higher than that of 200- and 300-μm treatments (*P* < 0.05).

**Table 2:** Effect of various corn particle sizes on the apparent total tract digestibility (ATTD) of nutrients in pigs under optimal thermal condition

Items	Particle size of corn (μm)					SE^1^
	200	300	400	600	800	
Intake						
Dry matter (g)	1058	1064	1063	1053	1018	56
Crude protein (g)	222	223	223	221	214	17
Ether extract (g)	77	78	78	77	74	4
Crude fiber (g)	35	35	35	35	34	3
Crude ash (g)	62	63	63	62	60	2
NDF (g)	94	95	95	94	91	3
ADF (g)	42.3	42	42	42	40	3
Energy (MJ/day)	22.4	22.6	22.5	22.3	21.6	0.7
Excretion (g)						
Dry matter	201^b^	233^a^	230^a^	224^ab^	213^ab^	10
Crude protein	38^b^	46^a^	46^a^	44^ab^	43^ab^	2
Ether extract	24^b^	30^a^	30^a^	30^a^	29^a^	1
Crude fiber	19	21	21	20	19	3
Crude ash	49^a^	47^ab^	46^ab^	46^ab^	44^b^	2
NDF	47	52	50	52	46	4
ADF	20	21	22	21	20	3
ATTD (%)						
Dry matter	81.00^a^	78.12^b^	78.30^b^	78.73^ab^	79.00^ab^	0.86
Crude protein	82.50^a^	79.02^b^	79.11^b^	79.81^b^	79.48^b^	1.01
Ether extract	68.12^a^	60.84^b^	60.93^b^	60.80^b^	61.00^b^	3.01
Crude fiber	44.50^a^	40.96^b^	40.27^b^	42.02^ab^	42.74^ab^	2.10
Crude ash	21.30^b^	23.73^b^	25.82^a^	25.82^a^	25.80^a^	1.98
NDF	48.86	44.29	46.50	44.01	48.70	3.56
ADF	50.38	50.25	48.20	49.88	49.33	2.65
DE^2^	82.61^a^	79.47^b^	79.25^b^	79.58^b^	79.39^b^	2.17
EUDE^3^	78.14^a^	74.07^b^	73.75^b^	74.32^b^	73.83^b^	2.30

^1^Standard error

^2^Digestible energy

^3^Excluding urinary energy loss in DE

^ab^Means in the same row with different superscripts differ (*P* < 0.05)

### Exp. 2. Under heat stress

Table 3 presents effects of various corn particle sizes on the apparent total tract digestibility of nutrients in heat stress conditions. Intakes of DM, CP, EE, CF, CA, NDF, ADF, and dietary energy from the 400-, 600-, and 800-μm diets were higher than those from the 200-μm diet (*P* < 0.05). Pigs fed the 800-μm diet had higher excretion of DM compared with those with the 200-μm diet (*P* < 0.05), and excretion of EE in 400-, 600-, and 800-μm diets was significantly higher than that of the 200-μm diet (*P* < 0.05). Apparent total tract digestibility of EE from 200- and 300-μm diets was higher than that of other treatments (*P* < 0.05). Contents of dietary energy in excretion from 400- and 800-μm diets were higher than those from 200- and 300-μm treatments (*P* < 0.05). The apparent total tract digestibility of energy was not significantly different among treatments (*P* > 0.05).

**Table 3:** Effect of various corn particle sizes on the apparent total tract digestibility (ATTD) of nutrients in pigs under heat stress

Items	Particle size of corn (μm)					SE^1^
	200	300	400	600	800	
Intake						
Dry matter (g)	896^b^	921^ab^	971^a^	991^a^	982^a^	41
Crude protein (g)	188^b^	194^ab^	204^a^	208^a^	206^a^	8
Ether extract (g)	66^b^	68^ab^	71^a^	73^a^	72^a^	1
Crude fiber (g)	30^b^	31^ab^	32^a^	33^a^	33^a^	1
Crude ash (g)	40^b^	41^ab^	44^a^	45^a^	44^a^	2
NDF (g)	68^b^	70^ab^	74^a^	75^a^	75^a^	2
ADF (g)	36^b^	37^ab^	39^a^	40^a^	39^a^	1
Energy (MJ/day)	19.0^b^	19.5^ab^	20.6^a^	21.0^a^	20.8^a^	0.5
Excretion (g)						
Dry matter	136^b^	145^ab^	150^ab^	149^ab^	153^a^	9
Crude protein	27	29	30	28	27	5
Ether extract	9^b^	11^b^	15^a^	17^a^	19^a^	3
Crude fiber	12	14	14	11	12	3
Crude ash	36	40	43	40	41	5
NDF	43^ab^	45^ab^	50^a^	39^b^	41^b^	4
ADF	18^b^	20^ab^	24^a^	18^b^	19^b^	1
ATTD (%)						
Dry matter	84.82	84.26	84.54	84.96	84.43	5.65
Crude protein	85.77	85.17	85.15	86.76	86.89	6.21
Ether extract	85.77^a^	83.53^a^	78.58^b^	76.70^b^	73.55^b^	3.02
Crude fiber	60.49^ab^	55.26^b^	55.61^b^	66.25^a^	64.61^ab^	4.01
Crude ash	11.62^a^	3.79^b^	2.12^b^	10.41^a^	7.94^a^	3.11
NDF	37.08^b^	35.16^b^	31.87^c^	47.55^a^	44.67^ab^	2.01
ADF	49.50^ab^	46.04^b^	38.52^c^	55.59^a^	51.35^a^	2.56
DE^2^	86.93	86.54	85.69	86.33	85.39	4.78
EUDE^3^	76.46	75.55	77.30	78.00	76.76	3.83

^1^Standard error

^2^Digestible energy

^3^Excluding urinary energy loss in DE

^abc^Means in the same row with different superscripts differ (*P* < 0.05)

## Discussion

### Exp. 1. Under optimal thermal conditions

In our study, pigs fed a diet of smaller particle size had greater nutrient digestibility under optimal thermal conditions. This result is consistent with the findings of Kim et al. (2002), who showed that pigs fed a diet of 500-μm particle size had significantly higher nutrient digestibility than those fed a diet of 1000-μm particle size. Paulk et al. (2011) reported a greater ADG for finishing pigs fed diets containing finely ground corn than for those fed on coarsely ground corn. Similarly, Wondra et al. (1995b) demonstrated that reducing the particle size of cereal grains to a minimum of 600 μm resulted in greater nutrient digestibility, rate of growth, and lactation performance, and decreased fecal excretion of nutrients, than was observed for grains of coarser particle size (from 900 to 1000 μm). Thus, the results of both the present and previous studies indicate that a smaller particle size of corn is beneficial for pig performance. Additionally, Kim et al. (2000) showed that corn of a smaller particle size had the most beneficial effect on apparent total tract digestibility of energy, which is again consistent with the results of the present study. Reduced particle size of grains has been reported to improve nutrient digestibility, growth rate, feed intake, feed conversion ratio, and gut health (Choct et al. 2004). As such, the results we obtained under optimal thermal conditions, showing that corn of smaller particle size has positive effects, are generally consistent with the findings of previous studies.

### Exp. 2. Under heat stress

In contrast to the optimal thermal conditions, overall in heavier pigs, the nutrient digestibility tended to decrease under heat stress conditions. The availability of amino acid for growth of pigs exposed to comfort-like ambient temperature may differ from that when pigs are exposed to heat stress within the same day (Cervantes et al. 2017). According to Le Bellego et al. (2002), there was lower protein retention at heat stress condition than optimal thermal condition when pigs were fed the same amount of energy. Heat stress changes the organism physiology, metabolism, and behavior to maintain homeostasis. In pigs, HS increases the intestinal temperature (Morales et al. 2015) as well as reduced the proliferation of intestinal cells (Sonna et al. 2002). Furthermore, Yu et al. (2010) found that heat treatment caused marked damage to the tips of the intestinal villi and induced epithelial cell shedding, exposure of the intestinal mucosa lamina propria, and shortening of villus height and crypt depth in the small intestine. These damaged intestinal environments can deteriorate nutrient utilization in pigs. Heat stress creates a bottleneck that slows pyruvate entry into the TCA cycle, which thus increases pyruvate-derived metabolite production. These results negatively contribute to the altered postabsorptive carbohydrate metabolism (Baumgard and Rhoads Jr 2013). For these reasons, it seems that the effects of reduction in particle size were not observed due to reduced nutrient digestibility. Mertens (1997) reported that feeding diets of larger particle size would increase salivary secretion, which would have promoted digestion by salivary amylase. Salivary amylase may well represent a potential compensatory alternative pathway for the digestion of amylose, amylopectin, and glycogen (Emanual 1987). Moreover, saliva is the most important oral fluid, and is critical for the preservation and maintenance of oral health (Edgar 1992). These results indicate that a reduction in particle size to less than 600 μm is not necessary under heat stress conditions. Moreover, finely ground grains have been implicated as a cause of stomach ulcers in growing-finishing pigs (Wondra et al. 1995a). The higher cell wall digestibility for the coarse diet than that for the fine diet could be related to rate of passage. In growing-finishing pigs, the cell wall particles of the coarse diet had a significantly longer retention time (Fioramonti and Bueno 1980; Ehle et al. 1982). Therefore, larger particle size, with a longer retention in the pig than smaller particle size, would be expected to show a greater extent of cell wall digestion. A diet ground too coarsely is potentially inefficiently utilized due to the rapid passage of the large particles through the digestive tract of the animal, resulting in ineffective or incomplete mastication and digestion of the diet (Ivan et al. 1974). In contrast, diets in which the particle size of grains is too fine would become more sticky and unpalatable in the mouth of pigs (Little 1997). Accordingly, on the basis of the various negative effects indicated by previous studies, it would be advantageous not to reduce the particle size of feeds under conditions of heat stress.

## Conclusion

In conclusion, for the lighter pigs, grinding corn to 200-μm corn particles reduced energy wastage and improved the positive effect on nutrient digestibility under optimal thermal condition; whereas for the heavier pigs, there was no beneficial effect on energy digestibility among treatments. Furthermore, in heavier pigs, the 600-μm diet had positive effects on digestibility of CF, NDF, and ADF than 200-μm corn particle size under heat stress conditions.
